# Electrolyte and Acid-Base Disorders Triggered by Aminoglycoside or Colistin Therapy: A Systematic Review

**DOI:** 10.3390/antibiotics10020140

**Published:** 2021-02-01

**Authors:** Martin Scoglio, Gabriel Bronz, Pietro O. Rinoldi, Pietro B. Faré, Céline Betti, Mario G. Bianchetti, Giacomo D. Simonetti, Viola Gennaro, Samuele Renzi, Sebastiano A. G. Lava, Gregorio P. Milani

**Affiliations:** 1 Faculty of Biomedicine, Università della Svizzera Italiana, 6900 Lugano, Switzerland; gabriel.bronz@hotmail.com (G.B.); po.rinoldi@gmail.com (P.O.R.); celine.betti@eoc.ch (C.B.); mario.bianchetti@usi.ch (M.G.B.); giacomo.simonetti@eoc.ch (G.D.S.); vio.gennaro94@live.it (V.G.); 2 Department of Pediatrics, Pediatric Institute of Southern Switzerland, Ospedale San Giovanni, Ente Ospedaliero Cantonale, 6500 Bellinzona, Switzerland; gregorio.milani@unimi.it; 3 Department of Internal Medicine, Ospedale La Carità, Ente Ospedaliero Cantonale, 6600 Locarno, Switzerland; pietrobenedetto.fare@eoc.ch; 4 Division of Hematology and Oncology, The Hospital for Sick Children, Toronto, ON M5G 1X8, Canada; samuele.renzi@sickkids.ca; 5 Pediatric Cardiology Unit, Department of Pediatrics, Centre Hospitalier Universitaire Vaudois, and University of Lausanne, 1011 Lausanne, Switzerland; sebastiano.lava@chuv.ch; 6 Pediatric Unit, Fondazione IRCCS Ca’ Granda Ospedale Maggiore Policlinico, 20122 Milan, Italy; 7 Department of Clinical Sciences and Community Health, Università degli Studi di Milano, 20122 Milan, Italy

**Keywords:** dyselectrolytemia, aminoglycosides, colistin, hypomagnesemia, hypokalemia, hypocalcemia

## Abstract

Aminoglycoside or colistin therapy may alter the renal tubular function without decreasing the glomerular filtration rate. This association has never been extensively investigated. We conducted a systematic review of the literature following the Preferred Reporting Items for Systematic Reviews and Meta-Analyses recommendations. Databases searched included United States National Library of Medicine, Excerpta Medica, and Web of Science. For the final analysis, we evaluated 46 reports, published after 1960, describing 82 cases. A total of 286 electrolyte and acid-base disorders were reported. Hypomagnesemia, hypokalemia, and hypocalcemia were reported in more than three quarter of cases. Further disorders were, in decreasing order of frequency, metabolic alkalosis, hyponatremia, hypophosphatemia, hypouricemia, hypernatremia, and metabolic acidosis. Six electrolyte and acid-base disorders were reported in seven cases, five in 12 cases, four in 16 cases, three in 31 cases, two in 11 cases, and one in five cases. Laboratory features consistent with a loop of Henle/distal tubular dysfunction were noted in 56 (68%), with a proximal tubular dysfunction in three (3.7%), and with a mixed dysfunction in five (6.1%) cases. The laboratory abnormality was unclassified in the remaining 18 (22%) cases. Treatment with aminoglycosides or colistin may trigger a proximal tubular or, more frequently, a loop of Henle/distal tubular dysfunction.

## 1. Introduction

Parenteral aminoglycoside antimicrobials are often prescribed for the treatment of infections caused by mycobacteria, Neisseria gonorrhoeae, and protozoa [1,2]. However, the most common application of these antimicrobials is for infections caused by aerobic Gram-negative bacteria [1]. Since many patients affected by cystic fibrosis have chronic pulmonary infection with Pseudomonas aeruginosa, many cystic fibrosis patients receive frequent and prolonged courses of these antimicrobials [2].

A mild renal impairment that is almost always reversible occurs in ≥10% of subjects receiving an aminoglycoside for a prolonged period [1]. On the other hand, the glycopeptide antibiotic colistin, which is used parenterally for infections caused by multi-drug resistant Gram-negative bacteria, possesses a nephrotoxicity similar to that of aminoglycosides [3].

It has been known for about 70 years that aminoglycoside therapy may sporadically alter the renal tubular function without decreasing the glomerular filtration rate [4]. Since this association has never been extensively investigated, we performed a systematic review of the published literature. The purpose of this review is to document the pattern of electrolyte and acid-base disorder and the time of resolution following aminoglycoside or colistin therapy.

## 2. Results

### 2.1. Search Results—Completeness of Reporting

**Figure 1:**
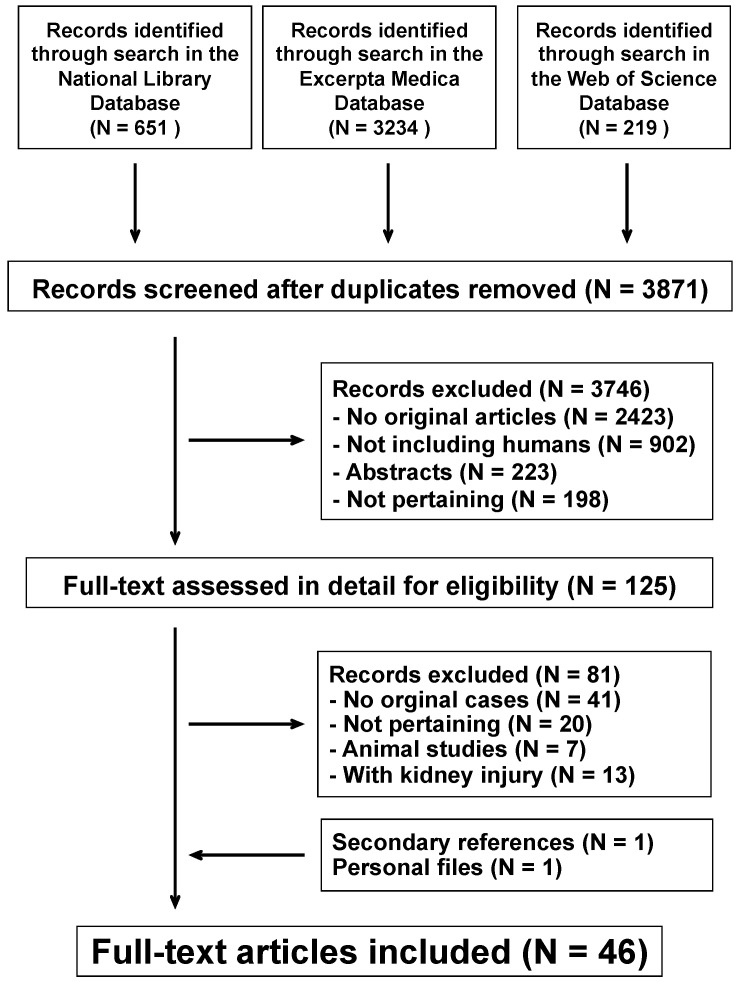
Flowchart of the literature search process.

The literature search process is summarized in Figure 1. For the final analysis, we evaluated 46 reports [5,6,7,8,9,10,11,12,13,14,15,16,17,18,19,20,21,22,23,24,25,26,27,28,29,30,31,32,33,34,35,36,37,38,39,40,41,42,43,44,45,46,47,48,49,50] published between 1961 and 2020 in English (N = 45) and Spanish (N = 1). They had been reported from the following countries: United States of America (N = 12), India (N = 8), United Kingdom (N = 7), Turkey (N = 4), Ireland (N = 3), Greece, Israel, Taiwan (N = 2), Australia, Belgium, Canada, Croatia, Paraguay, and Spain (N = 1). A total of 82 cases were identified among the articles reviewed, including 15 cystic fibrosis patients (nine females and six males 15 to 30 years of age). Reporting completeness was high in 31 (38%), moderate in 37 (45%), and low in the remaining 14 (17%) cases.

### 2.2. Findings

The baseline characteristics of the patients are listed in Table 1. Fifty-eight (71%) patients were ≥20 years of age, 19 (23%) were 3 to 19 years of age, three (3.7%) were 4 weeks to 2 years of age, and two (2.4%) < 4 weeks of age. Approximately half of the cases were non-tuberculous or tuberculous respiratory infections. The other common infections included abdominal, urinary, and drug-induced neutropenia; Aminoglycosides were prescribed in the vast majority of the 82 patients (most frequently gentamicin and tobramycin). The authors of the aforementioned reports never associated the observed electrolyte and acid-base disorders to an excessive aminoglycoside or colistin dosage. Thirty percent of the patients had at least one prior course of aminoglycoside treatment. Electrolyte and acid-base disorders were mostly (65%) reported ≤ 4 weeks after starting the antimicrobial treatment. Electrolyte and acid-base disorder was not clinically symptomatic in one third of the cases. Neuromuscular irritability or muscle weakness were observed in the remaining patients.

A total of 286 electrolyte and acid-base disorders were reported in the 82 patients (Table 2). Hypomagnesemia, hypokalemia, and hypocalcemia were reported in more than three quarter of cases. Proteinuria, glucosuria, and aminoaciduria were reported in <10% of cases. The urinary magnesium excretion, assessed in just over half of patients with hypomagnesemia, was always inappropriately high. Similarly, the urinary chloride excretion was inappropriately high in 21 cases with hypokalemic metabolic alkalosis.

Using the criteria suggested in the section, Materials and Methods, the patients were classified as shown in Table 3. Three quarters of the cases presented with a loop of Henle/distal tubular dysfunction (isolated or, more rarely, associated with a proximal tubular dysfunction) or with a proximal tubular dysfunction.

The time to resolution was ≤ 1 week in 10 (19%) cases, 2 to 4 weeks in 22 (41%) cases and > 4 weeks in 22 (41%) cases. This information was not available for 28 cases.

## 3. Discussion

The potential of aminoglycosides and colistin to induce renal tubular dysfunction has not been systematically addressed so far. This analysis demonstrates that treatment with aminoglycosides or colistin may transiently alter the renal tubular function, without any concomitant relevant decrease in glomerular filtration. We observed (1) a proximal tubular, (2) a loop of Henle/distal tubular, and (3) a mixed tubular dysfunction. However, we were not able to classify a minority of cases as proximal tubular, loop of Henle/distal tubular, or mixed tubular dysfunction. The clinical picture associated with the altered electrolyte and acid-base balance may include muscle weakness or neuromuscular irritability.

In this analysis, renal magnesium wasting was the most common electrolyte disorder. This observation is supported by the results of a well-designed study of patients treated with amikacin, where after two weeks, the only electrolyte abnormality noted was magnesium [51].

The mechanisms by which aminoglycosides may alter the renal tubular function remain speculative. Available data suggest that the proximal tubular diseases (and the acute kidney injury) might result from a mitochondrial dysfunction. On the other hand, the loop of Henle/distal tubular injury might result from an activation of the calcium-sensing receptor [52,53,54,55].

Several factors may modulate the potential of aminoglycosides to induce acute kidney injury including pre-existing reduced renal function, prolonged treatment, liver dysfunction, hypoalbuminemia, reduced effective blood volume, and combined therapy with other nephrotoxic drugs [1,2]. Finally, it is assumed that gentamicin has the greatest nephrotoxic potential, followed by tobramycin, amikacin, and netilmicin [1,2]. The results of the present analysis do not allow us to confirm if the information available for acute kidney injury may be extrapolated to renal tubular dysfunction.

In cystic fibrosis patients, hyponatremia, hypokalemia, and metabolic alkalosis may result either from a tubular disturbance or from excessive salt loss in sweat. Infants with cystic fibrosis are primarily at risk for this non-renal disturbance due to the poor salt content of infant formula and or breast milk. Likewise, in older cystic fibrosis patients, this biochemical abnormality may develop under heat stress. In contrast with renal tubular dysfunction, urinary chloride excretion is low in this setting [56,57].

A major strength of our systematic review validated that electrolyte and acid-base disorders can be triggered by aminoglycoside and colistin therapy. However, the current available information does not allow documenting the prevalence of such disorders associated with these antimicrobials. Second, the reporting completeness was high in <40% of cases. Future work is necessary to address this.

In conclusion, many classes of antimicrobials may cause electrolyte and acid-base disorders [58]. The present review supports the association of aminoglycosides and colistin therapy with either a proximal tubular or, more frequently, a loop of Henle/distal tubular dysfunction. In these patients, the glomerular filtration is normal or no more than marginally impaired.

## 4. Materials and Methods

### 4.1. Search Strategy

This review was accomplished following the Preferred Reporting Items for Systematic Reviews and Meta-Analyses recommendations [59]. Searches were run in the databases of the United States National Library of Medicine, Excerpta Medica, and Web of Science on July 10, 2020 and repeated on November 10, 2020. Original reports published after 1960, with no language limits considered. The search strategy incorporated the terms (acidosis OR alkalosis OR electrolyte disturbances OR hypercalcemia OR hyperkalemia OR hypermagnesemia OR hypernatremia OR hypocalcemia OR hypokalemia OR hypomagnesemia OR hyponatremia) AND (aminoglycoside OR amikacin OR capreomycin OR colistin OR dibekacin OR gentamicin OR isepamicin OR kanamycin OR neomycin OR netilmicin OR paromomycin OR plazomicin OR sisomicin OR spectinomycin OR [dihydro]streptomycin OR tobramycin OR viomycine). References listed within bibliographies of the retrieved records and pertinent personal files of the authors were also considered for inclusion.

Two authors independently screened all identified titles and abstracts in a nonblinded fashion. Upon retrieval of relevant reports, full-text publications were reviewed for eligibility.

### 4.2. Eligibility Criteria

We included original articles and letters that reported individual humans on parenteral aminoglycoside or colistin therapy presenting with otherwise unexplained electrolyte (total calcium ≥ 2.80 mmol/L; ionized calcium ≥ 1.40 mmol/L; total calcium ≤ 2.20 mmol/L; ionized calcium ≤ 1.10 mmol/L; total magnesium ≥ 1.20 mmol/L; total magnesium ≤ 0.70 mmol/L; sodium ≥ 146 mmol/L; sodium ≤ 134 mmol/L; potassium ≥ 5.1 mmol/L; potassium ≤ 3.4 mmol/L; inorganic phosphate ≥ 2.00 mmol/L; inorganic phosphate ≤ 1.00 mmol/L) or metabolic acid-base disorders (acidosis: bicarbonate ≤ 18 mmol/L and pH ≤ 7.38; alkalosis: bicarbonate ≥ 27 mmol/L and pH ≥ 7.42). Since children and adults importantly differ with respect to uric acid metabolism, age- and gender-dependent reference values were used for the definition of hyper- and hypouricemia [60].

Patients with preexisting conditions (alcohol use, diabetes, kidney disease), conditions predisposing to electrolyte and acid-base disorder (i.e., diarrhea, vomiting), concurrent agents with potential nephrotoxic activity (i.e., amphotericin B, cisplatin, diuretics, etc.), and finally those with a diagnosis of acute kidney injury (rise in circulating level to ≥1.5 times baseline or increase by ≥27 μmol/L above the upper limit of normal for age) were excluded [61]. Cystic fibrosis individuals with electrolyte abnormalities such as hyponatremia, hypokalemia, and metabolic alkalosis associated with low urinary chloride excretion were also excluded [56,57].

### 4.3. Data Extraction

Data were extracted using a piloted form and transcribed into a predefined spreadsheet. The data extracted from each case meeting inclusion criteria were demographics, diagnosis, clinical findings possibly associated with the electrolyte and acid-base disorder including neuromuscular irritability (such as perioral numbness, paresthesias of the hands and feet, muscle cramps, carpopedal spasm, laryngospasm, Chvostek sign, Lust sign, Trousseau sign, or seizures), muscle weakness or acidotic Kussmaul breathing, information on aminoglycoside (or colistin) therapy before onset of electrolyte or acid-base disorders, laboratory values, and time to resolution after withdrawing the antimicrobial therapy. Effort was made to retrieve following laboratory values: acid-base balance, levels of calcium, inorganic phosphate, magnesium, potassium, sodium or uric acid in blood, and renal parameters. Available information on glucosuria, aminoaciduria, and urinary excretion of magnesium or chloride was also recorded. If needed, attempts were also made to contact original authors to obtain missing data. The accuracy in describing diagnosis and clinical presentation, drug treatment, laboratory data, and time to recovery of electrolyte and acid-base imbalance was used to grade completeness of reporting as high, moderate, or low.

### 4.4. Analysis-Classification-Statistics

It has been hypothesized that electrolyte and acid-base disorders triggered by aminoglycoside therapy may result either from a proximal tubular or from an apparent loop of Henle/distal tubular dysfunction [58]. Hence, we tentatively made the diagnosis of proximal tubular dysfunction in patients presenting with at least three of the following: hypophosphatemia, hypouricemia, metabolic acidosis, proteinuria, glucosuria, or generalized aminoaciduria. On the other hand, we made the diagnosis of loop of Henle/distal tubular dysfunction in cases with at least three of the following: hypocalcemia, hypomagnesemia, hypokalemia, or metabolic alkalosis. The diagnosis of mixed tubular dysfunction was made in cases presenting with features consistent both with a proximal tubular and a loop of Henle/distal tubular dysfunction. The evaluation of urinary magnesium or chloride excretion was made as recommended in the literature [56]. The remaining cases were considered unclassified.

Missing data were managed by pairwise deletion. Categorical data are presented as frequency and were analyzed using the Fisher’s exact test. Continuous data are presented as median and interquartile range (≥6 cases) or as individual values (<6 cases) and were analyzed using the Kruskal–Wallis test and the Dunn posttest. Significance was assumed at *p* < 0.05.

## Figures and Tables

**Table 1:** Baseline characteristics of patients with electrolyte and acid-base disorder.

Characteristics	N	(%)
**Gender**		
Female	51	(62)
Male	31	(38)
**Underlying infection**		
Non-tuberculous respiratory infection *	23	(28)
Tuberculosis	17	(21)
Abdominal infection	13	(16)
Urinary tract infection	11	(13)
Infection associated with neutropenia ^☩^	5	(6.1)
Other	13	(16)
**Antimicrobial agent**		
Gentamicin	37	(45)
Tobramycin	11	(13)
Capreomycin	8	(9.8)
Kanamycin	4	(4.9)
Viomycin	2	(2.4)
Colistin	2	(2.4)
Unspecified aminoglycoside ^◆^	12	(15)
Other	6	(7.3)
**Previous antimicrobial therapy courses**	24	(29)
**Duration of antimicrobial therapy** **^✙^**		
≤1 week	11	(15)
2–4 weeks	37	(50)
2–6 months	17	(23)
>6 months	9	(12)
**Main findings**		
Neuromuscular irritability	42	(52)
Muscle weakness	2	(2.4)
Irritability and weakness	11	(13)

Data are presented as median (with interquartile range) or as frequency (with percentage) * 15 patients were affected by cystic fibrosis; ^☩^ induced by anticancer therapy; ^◆^ reference #24; **^✙^** information not available in 8 cases.

**Table 2:** Prevalence of electrolyte, acid-base disorder, and abnormal urinary findings.

Abnormal Finding	N	(%)
**Electrolyte and acid-base disorders**		
Hypomagnesemia	70	(85)
Hypokalemia	69	(84)
Hypocalcemia	64	(78)
Metabolic alkalosis	35	(43)
Hyponatremia	22	(27)
Hypophosphatemia	18	(22)
Hypouricemia	5	(6.1)
Hypernatremia	2	(2.4)
Metabolic acidosis	1	(1.2)
**Relevant urinary findings**		
Pathological proteinuria	6	(7.3)
Normoglycemic glucosuria	4	(4.9)
Generalized aminoaciduria	3	(3.7)
Inappropriately high electrolyte excretion		
Magnesium *	37	(45)
Chloride **	21	(26)

Data are presented as frequency (with percentage). * patients presenting with hypomagnesemia (24-h magnesium excretion ≥ 500 µmol or a fractional magnesium clearance ≥ 2.0 × 10^−2^); ** patients presenting with hypokalemia and metabolic alkalosis (24-h chloride excretion ≥ 20 mmol or fractional chloride clearance ≥ 0.5 × 10^−2^).

**Table 3:** Classification of electrolyte and acid-base disorders.

Disorder	N (%)	Females/Males	Age
Loop of Henle/Distal Tubular Dysfunction *	56 (68)	36/20	29 [19–57]
Proximal Tubular Dysfunction **	3 (3.7)	0/3	35, 71, 73
Mixed Dysfunction	5 (6.1)	3/2	41, 43, 49, 53, 64
Unclassified Abnormality	18 (22)	12/6	25 [19–28]

Data are presented as frequency, as median and interquartile range (≥6 cases), or as individual values (<6 cases). Patients with an unclassified electrolyte and acid-base disorder were significantly (*p* < 0.02) younger than patients with either and a mixed or a proximal tubular dysfunction. * hypomagnesemia, hypocalcemia, hypokalemia, or metabolic alkalosis; ** hypophosphatemia, hypouricemia, metabolic acidosis, proteinuria, glucosuria, or generalized aminoaciduria.

## References

[1] Craig W.A. (2011). Optimizing aminoglycoside use. Crit. Care Clin..

[2] Goetz D., Singh S. (2016). Cystic fibrosis: Respiratory system disease. Pediatr. Clin. N. Am..

[3] Javan A.O., Shokouhi S., Sahraei Z. (2015). A review on colistin nephrotoxicity. Eur. J. Clin. Pharmacol..

[4] Werner C.A., Tompsett R., Muschenheim C., McDermott W. (1951). The toxicity of viomycin in humans. Am. Rev. Tuberc..

[5] Clarke M., McCarthy C. (1961). Electrolyte changes due to viomycin. Tubercle.

[6] Holmes A.M., Hesling C.M., Wilson T.M. (1970). Capreomycin-induced serum electrolyte abnormalities. Thorax.

[7] Holmes A., Helsing C., Wilson T. (1970). Drug-induced secondary hyperaldosteronism in patients with pulmonary tuberculosis. QJM..

[8] Vanasin B., Colmer M., Davis P.J. (1972). Hypocalcemia, hypomagnesemia and hypokalemia during chemotherapy of pulmonary tuberculosis. Chest.

[9] Bar R.S., Wilson H.E., Mazzaferri E.L. (1975). Hypomagnesemic hypocalcemia secondary to renal magnesium wasting. Ann. Intern. Med..

[10] Patel R., Savage A. (1979). Symptomatic hypomagnesemia associated with gentamicin therapy. Nephron.

[11] Daele M.C.-V., Corbeel L., Van De Casseye W., Standaert L. (1980). Gentamicin-induced Fanconi syndrome. J. Pediatr..

[12] Watson A.J., McCann S.R., Temperley I.J. (1981). Tetany following aminoglycoside therapy. Ir. J. Med. Sci..

[13] Darr M., Hamburger S., Ellerbeck E. (1982). Acid-base and electrolyte abnormalities due to capreomycin. South. Med. J..

[14] Teziç T., Saraçlar Y., Bilginturan N., Kilcioğlu I. (1982). Symptomatic hypocalcemia and hypomagnesemia due to gentamicin therapy in an 8-year-old girl. Turk. J. Pediatr..

[15] Watson A., Coffey L., Keogh B., McCann S.R. (1983). Severe hypomagnesaemia and hypocalcaemia following gentamicin therapy. Ir. Med. J..

[16] Nanji A.A., Denegri J.F. (1984). Hypomagnesemia associated with gentamicin therapy. Drug Intell. Clin. Pharm..

[17] Watson A.J.S., Watson M.M.R., Keogh J.A.B. (1984). Metabolic abnormalities associated with tobramycin therapy. Ir. J. Med. Sci..

[18] Goodhart G.L., Handelsman S. (1985). Gentamicin and hypokalemia. Ann. Intern. Med..

[19] Green C.G., Doershuk C.F., Stern R.C. (1985). Symptomatic hypomagnesemia in cystic fibrosis. J. Pediatr..

[20] Davies S.V., Murray J.A. (1986). Amphotericin B, aminoglycosides, and hypomagnesaemic tetany. BMJ.

[21] Steiner R.W., Omachi A.S. (1986). A Bartter’s-like syndrome from capreomycin, and a similar gentamicin tubulopathy. Am. J. Kidney Dis..

[22] Wilkinson R., Lucas G.L., Heath D.A., Franklin I.M., Boughton B.J. (1986). Hypomagnesaemic tetany associated with prolonged treatment with aminoglycosides. BMJ.

[23] Kes P., Reiner Z. (1990). Symptomatic hypomagnesemia associated with gentamicin therapy. Magnes. Trace Elem..

[24] Fuchs S., Kaminski N., Brezis M. (1994). Drug points: Metabolic abnormality induced by streptomycin. BMJ.

[25] Melnick J.Z., Baum M., Thompson J.R. (1994). Aminoglycoside-induced Fanconi’s syndrome. Am. J. Kidney Dis..

[26] Slayton W., Anstine D., Lakhdir F., Sleasman J., Neiberger R. (1996). Tetany in a child with AIDS receiving Intravenous tobramycin. South. Med. J..

[27] Gainza F.J., Minguela J.I., Lampreabe I. (1997). Aminoglycoside-associated Fanconi’s syndrome: An underrecognized entity. Nephron.

[28] Landau D., Kher K.K. (1997). Gentamicin-induced Bartter-like syndrome. Pediatr. Nephrol..

[29] Adams J., Conway S., Wilson C. (1998). Hypomagnesaemic tetany associated with repeated courses of intravenous tobramycin in a patient with cystic fibrosis. Respir. Med..

[30] Akbar J.R.A., Rees J., Nyamugunduru G., English M.W., Spencer D.A., Weller P.H. (1999). Aminoglycoside-associated hypomagnesaemia in children with cystic fibrosis. Acta Paediatr..

[31] Liamis G., Alexandridis G., Bairaktari E.T., Elisaf M.S. (2000). Aminoglycoside-induced metabolic abnormalities. Ann. Clin. Biochem. Int. J. Lab. Med..

[32] Shetty A.K., Rogers N.L., Mannick E.E., Aviles D.H. (2000). Syndrome of hypokalemic metabolic alkalosis and hypomagnesemia associated with gentamicin therapy: Case reports. Clin. Pediatr..

[33] Alexandridis G., Liberopoulos E., Elisaf M. (2003). Aminoglycoside-induced reversible tubular dysfunction. Pharmacology..

[34] Chou C.-L., Chau T., Lin S.-H., Chen Y.-H. (2005). Acquired Bartter-like syndrome associated with gentamicin administration. Am. J. Med. Sci..

[35] Ghiculescu R.A., Kubler P.A. (2006). Aminoglycoside-associated Fanconi syndrome. Am. J. Kidney Dis..

[36] Chen Y.-S., Fang H.-C., Chou K.-J., Lee P.-T., Hsu C.-Y., Huang W.-C., Chung H.-M., Chen C.-L. (2009). Gentamicin-induced Bartter-like syndrome. Am. J. Kidney Dis..

[37] Chrispal A., Boorugu H., Prabhakar A.T., Moses V. (2009). Amikacin-induced type 5 Bartter-like syndrome with severe hypocalcemia. J. Postgrad. Med..

[38] Geara A.S., Parikh A., Rekhtman Y., Rao M.K. (2012). The case: Metabolic alkalosis in a patient with cystic fibrosis. Kidney Int..

[39] Çakır U., Alan S., Zeybek C., Erdeve Ö., Atasay B., Yalcinkaya F., Arsan S. (2013). Acquired Bartter-like syndrome associated with colistin use in a preterm infant. Ren. Fail..

[40] Varma T., Saini A., Panchani R., Gupta N.R. (2013). Two unusual cases of severe recalcitrant hypocalcemia due to aminoglycoside-induced hypomagnesemia. Indian J. Endocrinol. Metab..

[41] Sangsiraprapha W., Addison D., Longfield E., Workeneh B. (2013). A novel case of persistent Bartter’s-like syndrome associated with gentamicin exposure. Saudi J. Kidney Dis. Transplant..

[42] Sandal G., Akbay S., Ozen M. (2014). Acquired Bartter-like syndrome association with netilmicin therapy in an extremely low birth weight infant. Ren. Fail..

[43] Santra G., Paul R., Karak A., Mukhopadhay S. (2016). Gitelman-like syndrome with kanamycin toxicity. J. Assoc. Physicians India.

[44] Singh J., Patel M.L., Gupta K.K., Pandey S., Dinkar A. (2016). Acquired Bartter syndrome following gentamicin therapy. Indian J. Nephrol..

[45] Sharma P., Sahay R.N. (2017). Unusual Complication of multidrug resistant tuberculosis. Case Rep. Nephrol..

[46] Acosta A.G.R., Díaz A.V.A., Elias M.M.E.L., Vázquez C.A.V. (2018). Pseudo Bartter syndrome associated with intravenous infusion of colistin. Rev. Virtual Soc. Paraguaya Med. Interna.

[47] Puri M.M., Kumar A., Aneja P., Gupta R., Kumar L., Sarin R. (2019). Tetany in an extensively drug resistant tuberculosis (XDR-TB) patient treated with capreomycin. J. Assoc. Physicians India.

[48] Ravi C., Dabadghao P. (2019). Treatment of multi-drug resistant tuberculosis causing tubulopathy—Gitelman-like syndrome. Indian Pediatr..

[49] Yilmaz F., Nephrology A.A.S.H.C.O., Bora F., Ersoy F.F. (2019). Gentamicin-induced acquired Bartter-like syndrome: A case report and review of the literature. Turk. J. Nephrol..

[50] Veena E.R., Parrikar A., Keny S., Lawande D. (2020). Gitelman-like syndrome: A rare complication of using aminoglycosides in tuberculosis—A case report. Indian J. Tuberc..

[51] Von Vigier R.O., Truttmann A.C., Zindler-Schmocker K., Bettinelli A., Aebischer C.C., Wermuth B., Bianchetti M.G. (2000). Aminoglycosides and renal magnesium homeostasis in humans. Nephrol. Dial. Transpl..

[52] Zorov D.B. (2010). Amelioration of aminoglycoside nephrotoxicity requires protection of renal mitochondria. Kidney Int..

[53] Emma F., Salviati L. (2017). Mitochondrial cytopathies and the kidney. Néphrol. Thér..

[54] Hannan F.M., Kallay E., Chang W., Brandi M.L., Thakker R.V. (2018). The calcium-sensing receptor in physiology and in calcitropic and noncalcitropic diseases. Nat. Rev. Endocrinol..

[55] Saidak Z., Brazier M., Kamel S., Mentaverri R. (2009). Agonists and allosteric modulators of the calcium-sensing receptor and their therapeutic applications. Mol. Pharmacol..

[56] Mersin S.S., Ramelli G.P., Laux-End R., Bianchetti M.G. (1995). Urinary chloride excretion distinguishes between renal and extrarenal metabolic alkalosis. Eur. J. Pediatr..

[57] Scurati-Manzoni E., Fossali E.F., Agostoni C., Riva E., Simonetti G.D., Zanolari-Calderari M., Bianchetti M.G., Lava S.A.G. (2014). Electrolyte abnormalities in cystic fibrosis: Systematic review of the literature. Pediatr. Nephrol..

[58] Zietse R., Zoutendijk R., Hoorn E.J. (2009). Fluid, electrolyte and acid–base disorders associated with antibiotic therapy. Nat. Rev. Nephrol..

[59] Liberati A., Altman D.G., Tetzlaff J., Mulrow C.D., Gøtzsche P.C., Ioannidis J.P., Clarke M.F., Devereaux P.J., Kleijnen J., Moher D. (2009). The PRISMA statement for reporting systematic reviews and meta-analyses of studies that evaluate health care interventions: Explanation and elaboration. Ann. Intern. Med..

[60] Stiburkova B., Bleyer A.J. (2012). Changes in serum urate and urate excretion with age. Adv. Chronic Kidney Dis..

[61] Kellum J.A., Lameire N., Aspelin P., Barsoum R.S., Burdmann E.A., Goldstein S.L., Herzog C.A., Joannidis M., Kribben A., Levey A.S. (2012). Kidney Disease: Improving Global Outcomes (KDIGO) Acute Kidney Injury Work Group. KDIGO clinical practice guideline for acute kidney injury. Kidney Int..

